# Enhanced Image-Based Endoscopic Pathological Site Classification Using an Ensemble of Deep Learning Models

**DOI:** 10.3390/s20215982

**Published:** 2020-10-22

**Authors:** Dat Tien Nguyen, Min Beom Lee, Tuyen Danh Pham, Ganbayar Batchuluun, Muhammad Arsalan, Kang Ryoung Park

**Affiliations:** Division of Electronics and Electrical Engineering, Dongguk University, 30 Pildong-ro 1-gil, Jung-gu, Seoul 04620, Korea; nguyentiendat@dongguk.edu (D.T.N.); mblee@dongguk.edu (M.B.L.); phamdanhtuyen@dongguk.edu (T.D.P.); arsal@dongguk.edu (M.A.); parkgr@dongguk.edu (K.R.P.)

**Keywords:** pathological site classification, in vivo endoscopy, computer-aided diagnosis, artificial intelligence, ensemble learning

## Abstract

In vivo diseases such as colorectal cancer and gastric cancer are increasingly occurring in humans. These are two of the most common types of cancer that cause death worldwide. Therefore, the early detection and treatment of these types of cancer are crucial for saving lives. With the advances in technology and image processing techniques, computer-aided diagnosis (CAD) systems have been developed and applied in several medical systems to assist doctors in diagnosing diseases using imaging technology. In this study, we propose a CAD method to preclassify the in vivo endoscopic images into negative (images without evidence of a disease) and positive (images that possibly include pathological sites such as a polyp or suspected regions including complex vascular information) cases. The goal of our study is to assist doctors to focus on the positive frames of endoscopic sequence rather than the negative frames. Consequently, we can help in enhancing the performance and mitigating the efforts of doctors in the diagnosis procedure. Although previous studies were conducted to solve this problem, they were mostly based on a single classification model, thus limiting the classification performance. Thus, we propose the use of multiple classification models based on ensemble learning techniques to enhance the performance of pathological site classification. Through experiments with an open database, we confirmed that the ensemble of multiple deep learning-based models with different network architectures is more efficient for enhancing the performance of pathological site classification using a CAD system as compared to the state-of-the-art methods.

## 1. Introduction

Currently, cancer is a leading cause of human death worldwide [1]. There are several types of cancer such as lung [2,3], breast [4,5], skin [6,7], stomach [8,9,10], colorectal (also known as colon cancer) [11,12,13,14,15,16], thyroid [17,18,19], and brain [20,21,22] cancers. Among these, stomach and colorectal cancer (CRC) are two of the most common types causing death in humans. To diagnose these types of cancer, in vivo endoscopy is widely used. This technique allows for a detailed visualization of the in vivo structure of the colon or stomach, which is significantly useful to doctors for examining the evidence of disease. However, conventional medical imaging-based diagnostic techniques are still predominantly dependent on the personal knowledge and experiences of doctors (radiologists). Consequently, the diagnosis results have a large variance. To reduce this variance, a double-screening process can be invoked, in which two or more experts (radiologists) are required to read the captured medical images of a single case. Although this method is more efficient than the conventional diagnosis method, it is costly and time-consuming. Recently, a computer-aided diagnosis (CAD) has been widely employed to assist doctors in the diagnosis process, and it is now becoming important for enhancing the performance of the diagnosis process using medical imaging techniques.

Depending to the stage of the disease, several signatures may appear on the colon or stomach, such as polyp or gastritis regions. These regions are called pathological sites. Previous studies on pathological site detection/classification mostly used a single convolutional neural network (CNN). In a previous study [11], Patino-Barrientoo et al. proposed the use of a Visual Geometry Group (VGG)-based network for classifying endoscopic colon images into malignant and nonmalignant (benign) cases. Similarly, Ribeiro et al. [13] used the CNN for automated classification of polyp images. Experimental results with a relatively shallow network (five convolution layers and three fully connected layers) showed that the CNN is useful for polyp classification problems and outperforms other handcrafted feature extraction-based methods. Instead of training a new classification network, as shown in the studies by Patino-Barrientoo et al. [11] and Ribeiro et al. [13], Fonolla et al. [16] used a pretrained CNN model based on the residual network [23] that was trained on ImageNet image dataset as an image feature extractor, and classified input images into malignant and nonmalignant categories using conventional classification methods. Zhu et al. [9] also used a deep residual network and the transfer learning technique to determine the invasion depth of gastric cancer as well as the classification accuracy. They showed that the CNN-based method achieves significantly higher accuracy than human endoscopists. Similarly, Li et al. [10] used a CNN model, named GastricNet, for gastric cancer identification. As reported by their study, deep CNN-based methods are efficient for gastric cancer identification problems.

Although these studies showed that CNN-based methods have been successfully applied to solve the pathological site (polyp) classification problem, they all have a common limitation, which is the use of a single CNN model for the problem. As indicated by several previous studies on CNNs, the performance of the CNN-based method is highly dependent on several factors such as the depth and width of the network, number of network parameters, and architecture of the network. Among these factors, the design of network architecture performs an important role, especially when the depth of the network increases. All these previous studies used relatively shallow networks [11,13] or extracted image features at the last convolution layer of a deep residual network for classification problems [16]. As indicated by previous studies, the depth of a deep learning-based system can significantly enhance the performance of a detection/classification system, while a small number of network parameters help to prevent over/underfitting problems, ensuring that the network is easy to train [23,24,25,26,27]. Therefore, the performance of a classification system is limited because of the use of a shallow network or the extraction of image features using a pretrained model. In addition, it is significantly difficult to recognize pathological sites as benign or malignant cases at an early stage. Therefore, the classification of polyps into benign or malignant cases as performed by previous studies can yield incorrect results at the early stage of a disease. 

In a recent study, Kowsari et al. [28] proposed a new ensemble, deep learning approach for classification, namely random multi-model deep learning (RMDL). The RMDL approach solves the problem of finding the best deep learning structure and architecture to improve the classification. As a result of their study, the author showed that the ensemble learning method is efficient in enhancing the classification performance of various systems such as image-based system and text-based classification system. Inspired by the work by Kowsari et al., we proposed the use of multiple CNNs that differ in network architecture and depth to efficiently extract image texture features from input images to overcome these limitations of previous studies.

In contrast to previous studies that classify pathological site images into malignant and nonmalignant cases, our study classifies an input endoscopy image into one of two categories, with or without the appearance of pathological sites. Although this task can be accomplished by using a method to detect pathological sites in endoscopic images, the training process of a detection method requires strong efforts to accurately localize the pathological sites in the training dataset. However, at the early stage of the disease, the pathological sites are small and/or unclear, which can cause difficulties in creating ground-truth labels as well as in the detection process. Therefore, we simplified this task by simply classifying the input images into two classes (with or without the appearance of pathological sites) without the requirement for correct labeling of pathological site in input training images. This approach helps doctors focus on images with pathological sites rather than the other normal images during the diagnosis process. Table 1 lists comparative summaries of the proposed and previous studies. Our proposed method is novel in the following four ways:

- In a variation from the previous research focusing on only the polyp classification, our research is the first to classify pathological sites including both polyp and complex vascular information.
- To overcome the limitations of previous methods, whose performance is limited due to the use of a single model for classification, our study uses multiple deep learning-based models for pathological site classification. By employing an ensemble of multiple deep learning-based models, we can enhance the classification performance of pathological site classification.
- We employed and trained three different CNN model architectures, including VGG-, inception-, and densely connected convolutional network (DenseNet)-based networks, for ensemble learning purposes. Consequently, each model has its own strengths and weaknesses, and we can combine three models to enhance the classification performance using three combination methods, i.e., MAX, AVERAGE, and VOTING.
- Our algorithm is available to the public through [29] so that other researchers can impartially compare with our method.

The remainder of this paper is organized as follows. In Section 2, we propose an image-based pathological site classification method based on an ensemble of deep learning models. In Section 3, we present a validation of the performance of the method proposed in Section 2 using a public in vivo gastrointestinal (GI) endoscopic images, namely the in vivo GI endoscopy dataset [30], and compare it with previous studies and discuss our results. Finally, we present the conclusion of our study in Section 4.

## 2. Proposed Method

### 2.1. Overview of Proposed Method

**Figure 1:**
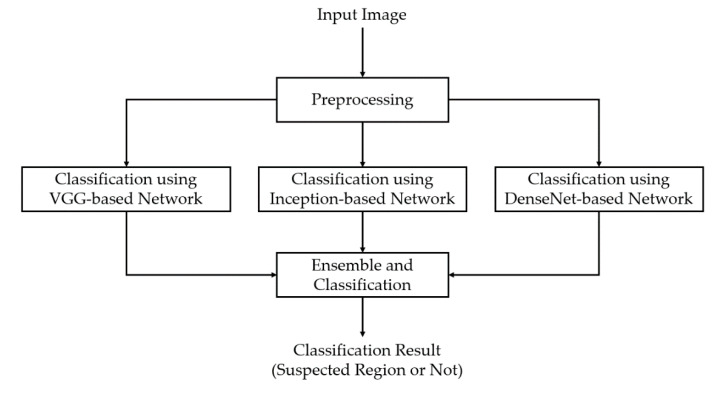
Overview of the proposed method.

Figure 1 shows the overall procedure of our proposed method for enhancing the performance of the pathological site classification system. As explained in Section 1, previous studies predominantly classified the pathological sites using conventional classification methods [11], or used a single CNN model [9,10,11,13,16]. Consequently, the classification performance is limited. These studies have a common drawback that they used single network architectures for classification problems. Therefore, the extracted image features are dependent on the network architecture. As stated in previous studies [23,24,25,26], the architecture of the CNN performs an important role in the performance of deep learning-based systems. For example, conventional CNNs, which are a linear stack of convolution layers, are suitable for a shallow design to solve a simple problem, whereas the residual or dense network is used to increase the depth of conventional CNNs; consequently, they can easily train a complex problem; alternatively, the inception network is used to extract richer features in a single network when compared to conventional CNNs. Based on these observations, we designed our CNN for the pathological site classification problem by incorporating these observations into a single classification network, as shown in Figure 1.

As shown in Figure 1, our proposed method first preprocesses the input endoscopic images to reduce noise and prepares them for inputting to the subsequent stages, which are based on deep learning techniques for the classification problem. A detailed explanation of this step is presented in Section 2.2. Our proposed method comprises of the following three main steps for classification. The first step is the classification using a conventional CNN based on VGG16 network architecture [25]. The second step is the classification using an inception-based network to extract richer features according to the different sizes of objects [26]; finally, the third step is based on a DenseNet architecture to exploit the effect of a significantly deep network [31]. From the results of these three networks, we can obtain three classification results. We believe that these three branches are equivalent to the previous studies that are based on a single simple CNN for classification problems. To enhance the performance of the pathological site classification problem, our study further combines these classification results using the ensemble learning technique and performs classification based on the combined results. These explanations are provided in detail in Section 2.3.

### 2.2. Preprocessing of Captured Endoscopic Images

**Figure 2:**
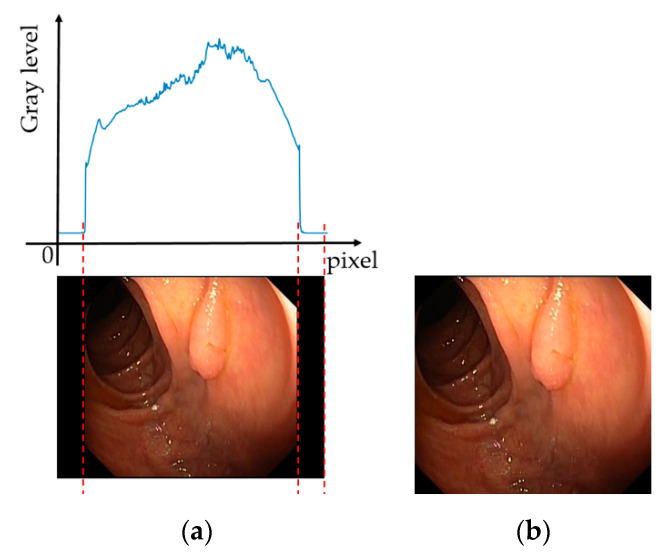
(**a**) Example of a captured gastrointestinal endoscopic image and (**b**) the corresponding result of the preprocessing step.

As explained in Section 2.1, the first step in our proposed method is the preprocessing step to eliminate redundant information from the endoscopic images before inputting them to CNNs. In Figure 2a, we show an example of a captured GI endoscopic image. As shown in this figure, the captured image normally contains two parts, including the background region with low illumination, and the foreground region with higher illumination. It can be observed that the background contains no useful information for our classification steps. Therefore, it should be removed before using the classification steps. This step is useful because the background region not only contains no information about the pathological sites but also presents noise that can consequently decrease the performance of further classification networks.

As shown in Figure 2a, the background region appears with a significantly low illumination when compared to the foreground region. Based on this characteristic, we implemented a simple method for background removal. A graphical explanation using a GI endoscopic image is shown in Figure 2a where the accumulated histogram-like features of pixels are represented in the horizontal direction. As shown in this figure, we first accumulate the histogram-like features of the input image by projecting the gray-level of the image pixel in the horizontal and vertical directions. Because of the low illumination characteristics of the background regions when compared to the foreground regions, we can arbitrarily set a threshold for separating the background and foreground. An example of the experimental result of this step using the image in Figure 2a is shown in Figure 2b. We can observe that, although Figure 2b still contains certain small background regions owing to the characteristics of the capturing devices, most of the background region was removed from the input image and the image is ready for further use in our proposed method.

### 2.3. Pathological Site Classification Method

#### 2.3.1. Deep Learning Framework

**Figure 3:**
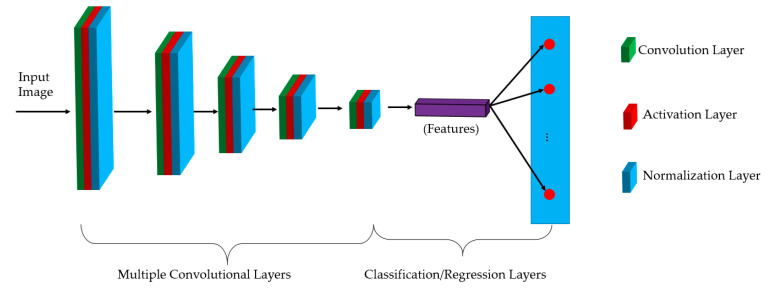
Conventional architecture of convolutional neural networks.

Recently, with the development of learning-based techniques, deep learning has been widely used in computer vision research. This technique has been successfully applied to various computer vision/pattern recognition problems such as image classification [23,24,25,26,27], object detection [32,33], and image generation [34,35,36]. The success of deep learning comes from the fact that this technique simulates the way in which the human brain processes information (images). Originally, a deep learning network was constructed by using several neural network layers to create a deep network that is used to process incoming information (images, voice, text, etc.). For computer vision research, an efficient type of deep learning technique, called CNN, has received significant attention. The idea of this type of deep learning technique is the use of convolution operations to extract useful information from input images, and the use of a neural network to efficiently solve a classification/regression problem. Figure 3 shows a graphical representation of a conventional CNN. As shown in this figure, a conventional CNN is composed of two primary parts, i.e., multiple convolution and classification/regression layers. The primary purpose of the convolution layers is to extract abstract and useful texture features that satisfactorily represent the characteristics/content of input images. The classification/regression layers are used to classify the input images (image classification problem), or regress continuous values such as height and width position of an object (object detection problem) based on the extracted image features produced by the first part of the CNN. Because of the use of convolution operation with the weight sharing scheme, the number of network parameters is significantly reduced when compared to a fully connected network with the same number of network layers. Consequently, it can help to successfully train a deep network and reduce the over/underfitting problem that normally occurs while using deep neural networks because of the large number of network parameters. However, with the increase in the depth and width of modern networks, the number of network parameters is still large, which prevents the successful training of a significantly deep network.

#### 2.3.2. Enhanced Convolutional Neural Network (CNN) Structures for Efficient Feature Extraction

As explained in Section 2.3.1, although the CNN method is efficient for many computer vision tasks, it still has several drawbacks that are caused by the presence of a large number of layers (depth) and network parameters. As the number of layers in the CNN increases, it increases difficulty in training the network and presents the gradient vanishing problem. In addition, training a network with a large number of network parameters requires a large amount of training data to prevent the over/underfitting problem. Further, extracting efficient texture features is also crucial for enhancing the performance of a CNN-based system. To reduce the effects of these problems and enhance the performance of CNN-based systems, several network architectures were proposed. In our study, we used two popular network architectures, including the inception and dense networks that are designed for this purpose.

**Figure 4:**
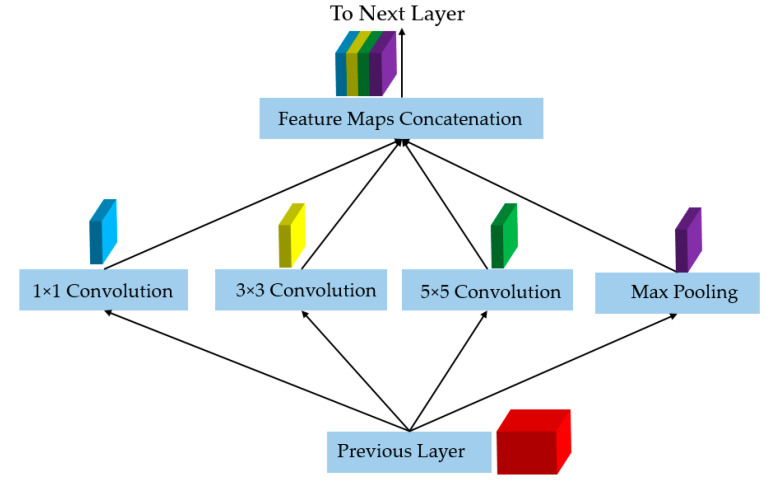
Naïve inception architecture for rich feature extraction used in inception network.

First, the conventional CNN is constructed by linearly stacking layers to create a deep network. Further, each convolution layer uses a fixed convolution kernel (such as 3 × 3, 5 × 5, or 7 × 7 kernel), as shown in Figure 3. However, when the problem becomes complex with the complex texture structure of input images and/or different sizes of objects, the use of fixed and single convolution kernels is insufficient for extracting efficient features for the classification/detection problem. To solve this problem, Szegedy et al. [26] proposed a new network architecture named inception block to extract richer information from input images than the conventional convolution layers. The concept of the inception block is depicted in Figure 4. In this figure, the input from the previous layer is shown as a red box, while the output and different feature maps obtained using various convolution kernels are marked as blue, yellow, green, and purple boxes. As shown in Figure 4, the inception block is constructed by using multiple convolution layers with different convolution kernels such as 1 × 1, 3 × 3, 5 × 5, and a max-pooling layer. These operations are performed in a parallel manner, and the results of all operations are concatenated to form the final input of an inception layer. It can be easily observed from Figure 4 that the inception layer can extract more useful texture information than the conventional convolution layer because of the use of different convolution kernels. A small convolution kernel can extract small texture features (small object texture). By increasing the convolution kernel, larger receptive fields are used to extract the texture information in the input images. By concatenating the results of all single convolution operations, the final feature maps are expected to contain a large amount of information at various texture levels when compared to the conventional convolution layer. Thus, more useful and efficient information is extracted using the inception layer. As demonstrated by the author of the inception method, it is significantly more efficient than a conventional CNN for the classification problem using the ImageNet dataset [26].

**Figure 5:**
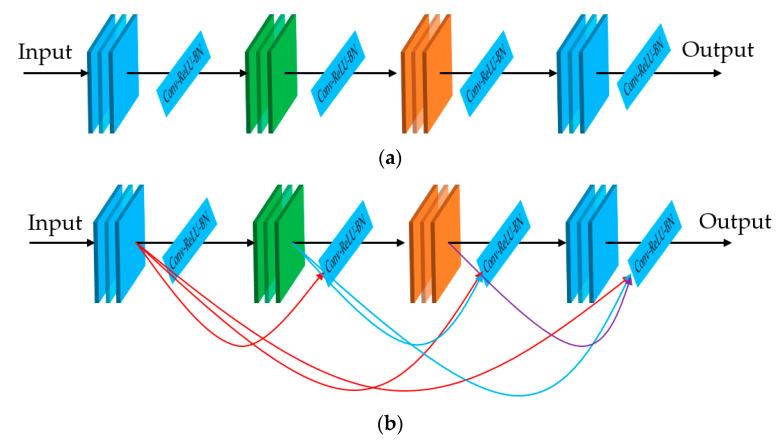
Comparison between (**a**) plain convolutional blocks and (**b**) dense-connection block architecture.

The second CNN architecture used in our study, the dense connection, was proposed by Huang et al. [31], which was designed to alleviate the vanishing gradient problem, reuse features, make the network easier to train, and reduce the number of network parameters. In Figure 5, we show the difference between the conventional CNN architecture (Figure 5a) and the dense-connection network architecture (Figure 5b). In this figure, Conv-ReLU-BN indicates the sequence of convolution (Conv), activation (rectified linear unit (ReLU)), and batch normalization (BN) blocks that are used to manipulate the input feature maps. While the conventional CNN architecture only has a connection between a single parent/child layer, the dense-connection architecture uses all the outputs of the preceding layers as inputs to the current layer. This design helps to reuse the features from early layers and reduces the effects of the vanishing gradient problem. By using dense connections, the network can be thinner and more compact. Consequently, it helps to reduce the number of network parameters that normally cause the over/underfitting problem in CNNs.

Inspired by the advantages of the inception and dense-connection networks, we used these two networks in addition to the conventional CNN based on the VGG architecture in our proposed method and experiments. Using these three networks with three different architectures, we processed input images in a different manner to enhance the performance of our classification system when combining the strength of each network.

#### 2.3.3. Ensemble of CNN Models for Pathological Site Classification

Based on the success of the CNN in the image classification problem, we propose a method for pathological site image classification, as shown in Figure 1 and Section 2.1. As explained in Section 2.3.1, although CNNs were successfully applied to various computer vision problems, this technique still has several drawbacks that prevent us from obtaining high-performance classification systems. First, the large number of network parameters can increase the difficulty in training the network and can also result in the over/underfitting problem. Second, the difference of the size of objects (texture features) that appear in the input images can affect the classification performance. To reduce the effects of these drawbacks and enhance the classification performance of the pathological site classification system, we propose the use of an ensemble learning technique that combines the classification results of three different CNN architectures for the problem, as shown in Figure 1.

In our study, we used three CNN architectures with different characteristics and depths for the classification problem, including VGG-based, DenseNet-based, and inception-based networks, as shown in Figure 1. Although it is possible to use other network architectures, we selected these network architectures because of our purpose for ensemble learning, that is, to combine the classification results of different network architectures in which each network classifies input images in a specific way. In our study, the VGG-based network serves as a conventional deep CNN for classification problems, which is composed of a linear stack of convolution layers. The DenseNet-based network serves as a very deep CNN with a short-cut path, which helps to easily train the network and extract more abstract and efficient image features. Further, the inception-based network helps extract image features with rich texture features and different sizes of objects. The detailed descriptions of these network architectures are listed in Table 2.

In this table, the convolution layers of the base networks (VGG16 [25], DenseNet121 [31], and inception [26] networks) are marked as “Main Convolution Layers.” For the classification part, we modified the number of output neurons from 1000 of the original network (the original networks were designed for the ImageNet classification challenge; therefore, it contains 1000 neurons at the output) to 2 neurons that represent two possible cases of our problem (with/without the appearance of pathological sites).

In the final step in our proposed method, as mentioned in Section 2.1, we attempted to combine the classification results of three different CNN models, i.e., VGG-, inception-, and DenseNet-based networks. In our study, we invoke three combination methods, including the MAX, AVERAGE, and VOTING rules, which are presented in Equations (1)–(3), respectively.

(1) $$\mathrm{MAX}\;\mathrm{rule}\;=argmax\left(\max\left(S_{i}\right)\right)$$

(2) $$\mathrm{AVERAGE}\;\mathrm{rule}\;=\;argmax\left(\frac{\sum_{i=1}^{n}S_{i}}{n}\right)$$

(3) $$\mathrm{VOTING}\;\mathrm{rule}\;=\;sign\left(\overset{n}{\underset{i=1}{\sum }}w_{i}\times argmax(S_{i})\right)$$

In Equations (1)–(3), $S_{i}$ indicates the classification probability of the *i^th^* classifier, and *n* indicates the number of classifiers. The “*argmax*” operator indicates the selection process of the class label whose classification probability is maximum. In our experiments, we used *n = 3* as we used three classifiers. As explained in the previous sections, we are dealing with a binary classification problem. Therefore, *Si* is a vector of two components, i.e.,$\;S_{i}=\left(S_{0i},S_{1i}\right)$, where $S_{0i}$ indicates the probability that the input image belongs to class 0 (negative cases (without pathological sites)), and $S_{1i}$ stands for the probability of the input image belonging to class 1 (positive case (with pathological sites)). In Equation (3), $w_{i}$ indicates the weight for the *i*th classifier and $argmax(S_{i})$ indicates the classification results of the *i*th classifier ($argmax(S_{i})=0$ for predicting the input image belonging to class 0; and $argmax(S_{i})=1$ for predicting the input image belonging to class 1. The weight value ($w_{i}$) is determined using Equation (4). In our experiments, we will measure the performance of the classification system using all three combination rules and select the rule that is most suitable with our pathological site classification problem based on the experimental results, as shown in Section 3.

(4) $$w_{i}=\;\{\begin{array}{c} -1\;if\;argmax(S_{i})\;=\;0\\ +1\;if\;argmax(S_{i})\;=\;1 \end{array}$$

### 2.4. Classification Performance Measurement

To measure the performance of a CAD system, previous studies used three popular metrics: Sensitivity, specificity, and overall accuracy [37,38]. These metrics are used to measure three different aspects of a binary classification system, i.e., the classification/detection ability of the system with respect to the positive (with the appearance of disease), negative (without the appearance of disease), and overall cases. The definition of these metrics is presented in Equations (5)–(7). In these equations, Sens, Spec, and Accuracy stands for sensitivity, specificity, and overall accuracy; TP indicates the number of true-positive cases (the case in which a positive case is correctly classified as a positive case); FN indicates the number of false-negative cases (the case where a positive case is incorrectly classified as a negative case); TN indicates the number of true-negative cases (the case in which a negative case is correctly classified as a negative case); finally, FP indicates the number of false-positive cases (the case in which a negative case is incorrectly classified as a positive case).

(5) $$\mathrm{Sens}=\;\frac{\mathrm{TP}}{\mathrm{TP}+\;\mathrm{FN}}$$

(6) $$\mathrm{Spec}=\;\frac{\mathrm{TN}}{\mathrm{TN}+\;\mathrm{FP}}$$

(7) $$\mathrm{Accuracy}=\;\frac{\mathrm{TP}+\mathrm{TN}}{\mathrm{TP}+\mathrm{TN}+\mathrm{FP}+\mathrm{FN}}$$

As indicated in Equation (5), the sensitivity measurement is the ratio between the TP samples over the total number of positive samples (TP + FN). Therefore, it indicates the ability of the classification system to detect positive cases (distinguish disease cases from all possible disease cases). The specificity is the ratio between the TN samples and the total number of negative samples. Consequently, the specificity is the measurement of the classification performance in detecting negative samples from all possible negative samples. Finally, the overall accuracy is measured by measuring the ratio between all correct classification samples (TP + TN) over all testing samples (TP + TN + FP + FN). Thus, the overall accuracy indicates the ability of a classification system to detect correct samples from the universe of samples.

In our study, we used these three measurements to evaluate the performance of our proposed method. In addition, as indicated by the meaning of the overall accuracy, we used the overall accuracy measurement to compare the performance of our proposed method with that of previous studies.

## 3. Experimental Results

Using the proposed method mentioned in Section 2, we conducted various experiments using the publicly available in vivo GI endoscopy dataset [30] to measure the classification performance of our proposed method in this section. The detailed experimental results are presented in the following subsections.

### 3.1. Dataset and Experimental Setups

**Figure 6:**
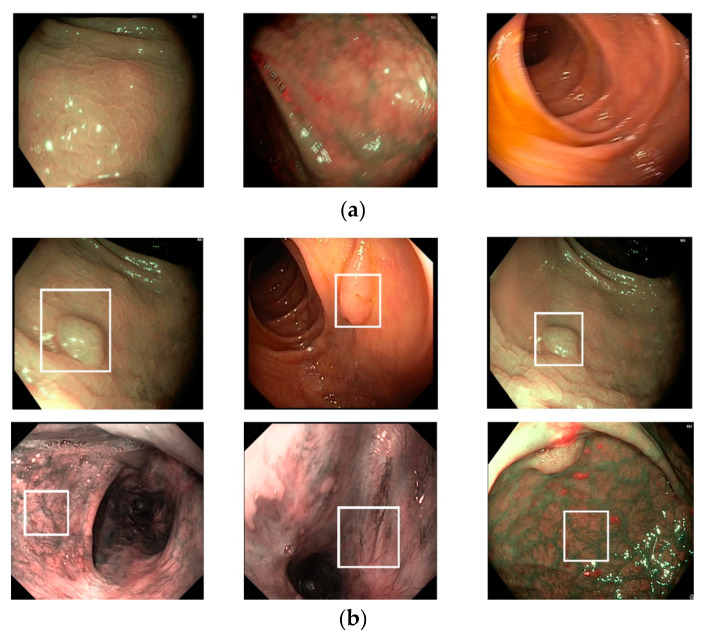
Example of images: (**a**) Gastric images without the existence of pathological sites and (**b**) gastric images with the existence of pathological sites (Upper and lower images indicate those including polyp and complex vascular regions, respectively).

To evaluate the performance of our proposed method and compare it with previous studies, we conducted experiments using a public dataset, namely the in vivo GI endoscopic dataset [30]. We called this dataset Hamlyn-GI for convenience, as this dataset is collected and provided by the Hamlyn Center for Robotic Surgery [30]. This dataset was originally collected for tracking and retargeting of GI endoscopic pathological sites using Olympus narrow-band imaging and Pentax i-Scan endoscope devices [30]. Specifically, this dataset contains 10 video sequences of GI endoscopic scans. Each video is saved in the format of successive still images. In the study by Ye et al. [30], the authors first manually defined a pathological site (a small polyp or suspected region) at the beginning of the endoscopic image sequence. Then, they tracked and retargeted this region for the remaining sequence of images. The information regarding the selected region and ground-truth tracked-retargeted region is provided for each video sequence in an annotation file. Because these regions are carefully set by experts and the polyp region or possible polyp regions are focused on, we used the information in the annotation file as an indicator of the existence of pathological sites in the still images. In the case of a still image containing a pathological site region, an approximate location of the pathological site is provided in the annotation file; otherwise, a negative value is provided. Based on the provided information in the annotation file, we preclassified the still images into two categories: With and without the existence of the pathological site in the stomach; that is, if the annotation of a still image is provided, then the still image is considered to contain the pathological site and assigned to the “with pathological site” class; otherwise, the still image is assigned to the “without pathological site” class. In Figure 6, we show certain examples of images from the Hamlyn-GI dataset. Figure 6a shows example images without the existence of pathological sites, whereas Figure 6b shows images containing pathological sites, which are marked with white bounding boxes. From Figure 6b, it can be observed that the bounding boxes are approximately provided by the author of the dataset. Therefore, they do not fit the correct location of pathological site regions. Consequently, it is difficult to perform a detection to solve this problem. Instead, we classified the input still images of this dataset into two classes: With and without the existence of pathological sites. In Table 3, we list the detailed statistical information regarding the Hamlyn-GI dataset. In total, the Hamlyn-GI dataset contains 7894 images.

To measure the performance of the proposed method, we performed two-fold cross-validation. For this purpose, we divided the Hamlyn-GI dataset into two separate parts, namely training and testing datasets. In the first fold, we assigned images of the first five video sequences (video files 1–5 in Table 3) as the training dataset and the images of the remaining five video sequences (video files 6–10 in Table 3) as the testing dataset. In the second fold, we exchanged the training and testing datasets of the first fold, i.e., training dataset contains images of the last five video sequences (video files 6–10 in Table 3) and the testing dataset contains images of the first five video sequences (video files 1–5 in Table 3). This division method ensures that the images of the same person (identity) only exist in either the training or testing dataset. Finally, the overall performance of the dataset with a two-fold cross-validation approach is measured by calculating the average (weighted by the number of testing images) of the two folds. In Table 4, we list a detailed description of the training and testing datasets in our experiments. In this table, “With PS” indicates the existence of a pathological site condition; further, “Without PS” indicates the absence of the existence of a pathological site condition. Although it is possible to use other cross-validation methods such as three-fold, five-fold, or leave-one-out approaches, we decided to use a two-fold approach in our experiments to save the processing time of the experiments.

### 3.2. Training

In our first experiment, we performed a training process to train the three deep learning-based models which are illustrated in Figure 1 and Table 2. For this experiment, we programmed our network using Python programming language with the help of the Tensorflow library [39] for the implementation of deep learning-based models. A detailed description of the parameters is listed in Table 5. For the training method, we used the adaptive moment estimation (Adam) optimizer with an initial learning rate of 0.0001, and we trained each model with 30 epochs. As the epoch increases, the network parameters become finer; therefore, we continuously reduced the learning rate after every epoch. In addition, a batch size of 32 was used in our experiment.

**Figure 7:**
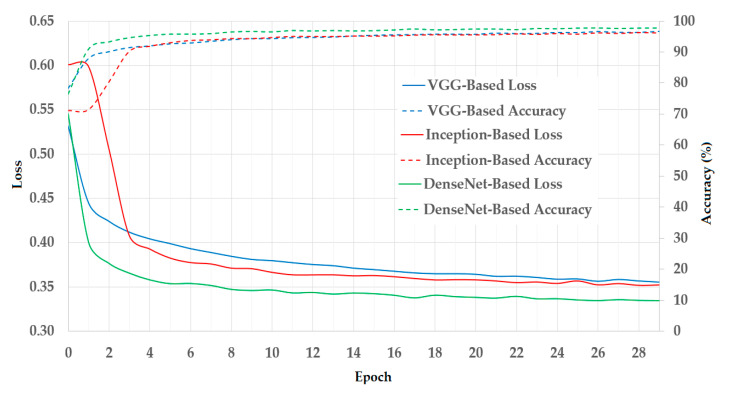
Training results (loss vs. accuracy) of Visual Geometry Group (VGG)-based, Inception-based, and DenseNet-based models in our experiments.

In Figure 7, we illustrated the results of the training process using the training datasets. As mentioned in Section 3.1, we used a two-fold cross-validation procedure in our experiments. Therefore, we calculated the average result of the two folds and presented it in Figure 7. In this figure, we show the curves of the loss and training accuracy of the training procedure for all three CNN models. As shown in these curves, the losses continuously decrease while the training accuracies increase with the increase in the training epoch. Thus, we can consider that the training procedures were successful in our experiments.

### 3.3. Testing of Proposed Method (Ablation Studies)

For the ablation studies, we performed the experiments presented in Section 3.3.1, Section 3.3.2 and Section 3.3.3.

#### 3.3.1. Classification Results Based on Individual CNN Models with the Preprocessing Procedure

As explained in Section 2 and Figure 1, our proposed method is composed of three main classification branches. Based on this structure, in our first experiment, we performed experiments using a single CNN-based classification method, as shown in Figure 1, using the three classification networks described in Table 2. For this experiment, we scaled the output images of the preprocessing step to 224 × 224 pixel-sized images and inputted them to each individual network for classification purposes. The detailed experimental results are listed in Table 6. From this table, it can be observed that the VGG-based network achieved an overall classification accuracy of 68.912% with a sensitivity of 66.271% and specificity of 71.946%. Similarly, we obtained an overall classification accuracy of 66.505% with a sensitivity of 87.438% and specificity of 42.476% using the inception-based network; moreover, an overall classification accuracy of 52.609% with a sensitivity of 19.554% and specificity of 90.558% was achieved using the DenseNet-based network. These experimental results show that the CNN is suitable for pathological site classification problems. However, the overall classification result was still low as the largest accurate classification result was obtained using a VGG-based network, which was measured to be 68.912%. This problem is caused by the fact that the dataset used in our experiments was not large and included the complex structure of the GI endoscopic images. In addition, we can observe from these experimental results that the VGG-based network outperforms the inception- and DenseNet-based networks. This is because the inception- and DenseNet-based networks are deeper than the VGG-based network. Consequently, the training of the network is affected because our dataset is not significantly big with images of only five patients.

We can also observe from Table 6 that the sensitivity and specificity measurements vary according to each CNN-based model. Using the VGG-based model, we obtained a sensitivity of 66.271% and specificity of 71.946%. This result indicates that the VGG-based network is more efficient in detecting negative images (images without possible pathological sites) than positive images (images with possible pathological sites). Similarly, we obtained a sensitivity of 19.554% and specificity of 90.558% using the DenseNet-based network. As it has a large value of specificity, the DenseNet-based network is more efficient in classifying negative images than positive ones. However, the situation is different when using the inception-based network. Using the inception-based network, we obtained a sensitivity of 87.438% and specificity of 42.476%. The high value of sensitivity indicates that the inception-based network is more efficient for classifying positive images than negative images. In addition, the difference between the sensitivity and specificity using the VGG-based network is approximately 5.675%, which is significantly smaller than the difference of 44.962% obtained using the inception-based network and 71.004% obtained using the DenseNet-based network. This result indicates that the VGG-based network has minimal bias while classifying positive and negative images when compared the other networks. This is because the VGG-based network is significantly shallower than the inception- and DenseNet-based networks. Therefore, the negative effects caused by the over/underfitting problem are less significant than those caused by the inception- and DenseNet-based networks.

#### 3.3.2. Classification Results Based on Individual CNN Models without the Preprocessing Procedure

As shown in Figure 1, our proposed method performs a preprocessing step before applying the classification step by using deep learning-based models to reduce the effect of noise on the classification results. In this experiment, we demonstrated the effects of noise and the advantages of the preprocessing step on our system by measuring the classification performance in a system that does not consider the preprocessing step. For this purpose, we trained and evaluated the performance of the deep learning-based models without considering noise reduction by the preprocessing step. The detailed experimental results are listed in Table 7. As shown in this table, we obtained overall classifications accuracies of 67.392%, 47.745% and 52.356% using the VGG-, inception-, and DenseNet-based methods, respectively. When compared to the experimental results in Table 6, the preprocessing procedure helps to enhance the classification results in the case of all three CNN models. Specifically, the classification accuracy is reduced from 68.912% in the case of using a preprocessing step to 67.392% in the case where a preprocessing step is not used in the experiment with the VGG-based method. In the case of the inception-based method, the overall classification accuracy is significantly reduced from 66.505% to 47.745% for the cases with and without the preprocessing step, respectively. Finally, a marginal reduction is observed in the case of the DenseNet-based method with an overall classification accuracy of 52.609% and 52.356% for the cases with and without the preprocessing step, respectively. Through these experimental results, we can observe that the background and noise have strong negative effects on the classification accuracy of the pathological site classification problem, and the preprocessing step is efficient in reducing these negative effects.

#### 3.3.3. Classification Results of the Proposed Ensemble Model of Three CNNs with the Preprocessing Procedure

As listed in Table 6, the three CNN-based models work differently with the same dataset. This indicates that each network has its own advantages and disadvantages for our classification problem. Based on these characteristics, we considered a combination of the results of these networks to enhance the performance of our classification system. Then, we performed an experiment to evaluate the performance of our proposed method mentioned in Section 2.1 using Equations (1)–(3). The detailed experimental results are listed in Table 8 for all three combination methods.

In the first experiment in this section, we used the MAX combination rule to combine the classification results of the three CNN-based models. As explained in Equation (1), the classification is performed based on the maximum classification scores of all three networks. From Table 8, it can be noted that the overall classification result (the average result of two folds) is 64.630% with a sensitivity of 57.952% and specificity of 72.299%. We can observe that this classification result is higher than 52.609%, which was obtained using only the DenseNet-based model (as listed in Table 6). However, this classification accuracy is lower than 68.912% and 66.505%, which were obtained using the VGG- and inception-based networks, respectively. As the specificity of 72.299% is higher than the sensitivity of 57.952%, we can conclude that the combined system based on the MAX rule is more efficient in recognizing the negative images than the positive images. In addition, the difference between the sensitivity and specificity using the MAX rule is approximately 14.347% (72.299–57.952%), which is smaller than the case of using only the inception- or DenseNet-based network. 

In the second experiment, we used the AVERAGE combination rule to combine the classification results of the three CNN-based models. As indicated by its meaning, the classification based on the AVERAGE rule was performed by classifying images based on the average classification scores of the three CNN-based models for negative and positive classes. As listed in Table 8, the AVERAGE combination rule produces an overall classification accuracy of 70.775% with a sensitivity of 68.452% and specificity of 73.442%. When compared to the classification accuracies listed in Table 6, we can observe that the classification accuracy by the AVERAGE rule is significantly better than that of the individual CNN-based models. The overall classification accuracy of 70.775% is significantly higher than the results of 68.912%, 66.505%, and 52.609% obtained using the VGG-, inception-, and DenseNet-based networks, respectively. In addition, the difference between the sensitivity and specificity of the AVERAGE rule is approximately 4.99% (73.442–68.452%), which is better than the value of 5.675% (the smallest difference between sensitivity and specificity of individual CNN-based models, as listed in Table 6) produced by the VGG-based network. This result indicates that the AVERAGE combination methods have minimal over/underfitting effects when compared to the individual CNNs. 

Finally, we performed an experiment using the VOTING combination rule, as indicated using Equations (3)–(4). As listed in Table 8, the VOTING combination rule produces an overall classification accuracy of 70.369% with a sensitivity of 68.452% and specificity of 72.571%. It can be observed that this overall classification accuracy is higher than the overall classification accuracy produced by the individual CNN-based models listed in Table 6. Although the overall classification accuracy produced by the VOTING rule is marginally lower than that produced by the AVERAGE rule (70.369% versus 70.775%), the difference is insignificant (approximately 0.406%). In addition, the difference between the sensitivity and specificity produced by the VOTING rule is 4.119% (72.571–68.452%). This value is the smallest value for the difference between the sensitivity and specificity and it indicates that the VOTING combination rule has fewer effects on the over/underfitting problem among the three combination rules and three individual CNN-based models.

**Figure 8:**
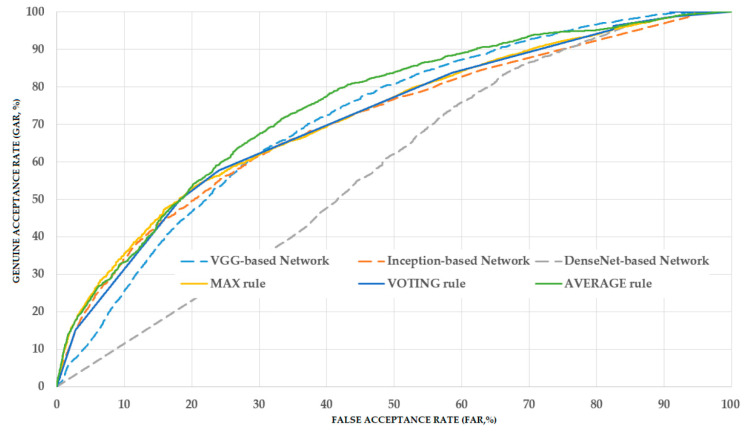
Receiver operating characteristic (ROC) curves of various system configurations in our experiment.

To deeply analyze the obtained experimental results, we obtained the receiver operating characteristic (ROC) curve of various system configurations, including three systems based on three individual CNN networks and three systems based on three combination rules, as shown in Figure 8. ROC curve is one of the most popular measurements used in medical statistic test, which provides a visualization of a classification performance. The ROC curve demonstrates the change of false acceptance rate (FAR) versus false rejection rate (FRR). In our experiment, we use the genuine acceptance rate (GAR) instead of FRR in measuring the ROC curve. That is, GAR is measured by (100 − FRR (%)). Because of this measurement method, a ROC curve looks like the ones in Figure 8, and the higher position on the upper-left side indicate the high performance of a classification system. As we perform a two-fold cross validation procedure, the ROC curves of each system configuration are obtained by taking average of the two ROC curves of two folds. From Figure 8, we can see that the AVERAGE rule outperforms the other system configurations, while the MAX and VOTING rule performs similar with the VGG-based or Inception-based network, and better than DenseNet-based network.

These experimental results show that the combination of the three CNN-based models is efficient in enhancing the classification performance of pathological site classification for a GI endoscopic examination when compared to previous studies. In addition, the AVERAGE and VOTING rules are more efficient than the MAX combination rule. As the best accuracy was obtained using the AVERAGE rule, we can conclude that the AVERAGE rule is the most suitable combination rule for implementation with the Hamlyn-GI dataset. However, as the size of the Hamlyn-GI dataset is relatively small, the applicability of these rules should be further investigated using larger data.

### 3.4. Comparisons with the State-of-the-Art Methods and Processing Time

Based on the experimental results presented in the above sections, we compared the classification performance of our proposed method with those of previous studies. As explained in Section 1, most of the previous studies used the conventional CNN (training a single shallow network or extracting image features from a pretrained network) for classification purposes. As discussed in Section 2.3 and Section 3.3, our proposed method applies the ensemble learning technique to three individual deep CNNs, i.e., VGG-, inception-, and DenseNet-based networks. Therefore, we can consider that the performances of previous studies correspond to the case of using each individual network. Based on this assumption, we conducted a comparison experiment and its results are listed in Table 9. From the table, it can be observed that by using the individual CNNs, we obtained the overall classification accuracies of 68.912%, 66.505% and 52.609% using the VGG-, inception-, and DenseNet-based networks, respectively. The best accuracy of 68.912% was obtained using the VGG-based method. Using our proposed method, we obtained the best accuracy of 70.775% with the AVERAGE combination rule, which is higher than the accuracy produced by the use of an individual network. Moreover, our proposed method with the VOTING combination rule also produced an accuracy of 70.369%, which is also higher than the best value of 68.912% of the individual network. From these experimental results, we can conclude that our proposed method outperforms previous studies in the pathological site classification system. However, as the best accuracy is still low (70.775%), the classification system still requires enhancements in our future works.

In the final experiment, we measured the processing time of our proposed method for pathological site classification problems. For this purpose, we created a deep CNN program in Python programming language using the Tensorflow library [39]. To run the program, we used a desktop computer with an Intel Core i7-6700 central processing unit with a working clock of 3.4 GHz and 64 GB of RAM memory. To increase the speed of the deep learning networks, we used a graphical processing unit, namely GeForce Titan X, to run the inference of the three deep learning models [40]. The detailed experimental results are listed in Table 10. We also performed the comparisons of the processing time between the proposed method and the previous studies. As shown in Table 10, it takes about 37.646 ms, 67.472 ms and 65.901 ms to classify an input image using VGG-based [11,25], Inception-based [26], and DenseNet-based [31] network, respectively. Using our proposed method, it takes 179.440 ms to classify a single input image, and our proposed method can work at a speed of 5.6 frames per second. From these experiment results, we can find that our proposed method takes longer processing time than previous studies. However, it is acceptable in medical image processing applications where the accuracy, but not processing time, is the primary requirement.

### 3.5. Analysis and Discussion

As shown in our experimental result, the average rule outperforms the max and voting rule in enhancing classification performance. This is caused by the fact that the average rule uses the prediction scores of individual CNN models directly, whereas the max rule is performed based on only the maximum classification score of one CNN model among several CNN models, and the voting rule is performed based on majority decisions of CNN models. As a result, the combined classification score by average rule contains more detailed classification information from all CNN models than the other two combination rules.

We used a public dataset, namely Hamlyn-GI, which showed the large visual differences among the collected images [30]. To measure the performance of the proposed method, we first divided the entire Hamlyn-GI dataset into training and testing sets. The division is done by ensuring that the training and testing datasets are different, i.e., images of the same patient only exist in either training or testing datasets as open world evaluation. As a result, these training and testing set are independent from the other as shown in Figure 6 (for example, the left-bottom and middle-bottom images are the samples of training data whereas the others are those of testing one for the 1st fold validation). We used the training dataset to train the classification models, and testing dataset to measure the performance of the trained models. Therefore, the measured performance is the optimal generalization because the testing set is independent from the training set.

**Figure 9:**
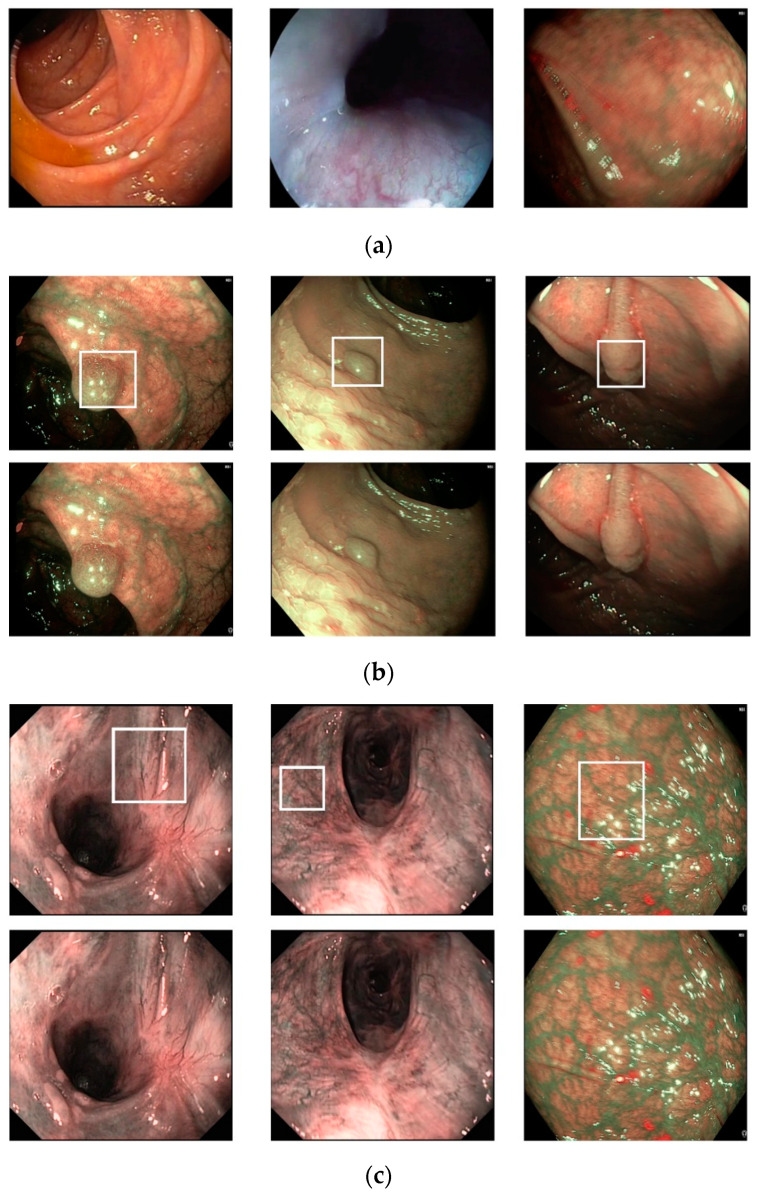
Examples of correct classification results by the proposed method: (**a**) True-negative cases and (**b**,**c**) true-positive cases (Upper and lower images indicate the ground-truth and testing images, respectively).

**Figure 10:**
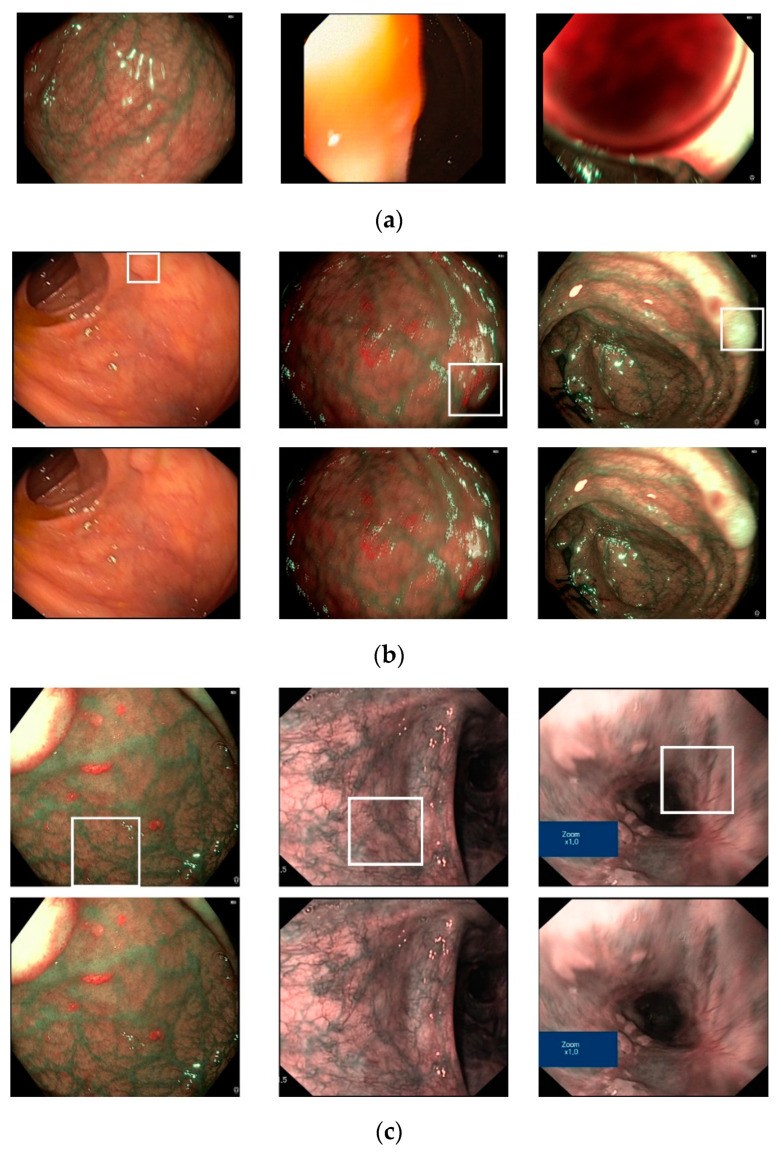
Examples of incorrect classification results by the proposed method: (**a**) False-positive cases and (**b**,**c**) false-negative cases (Upper and lower images indicate the ground-truth and testing images, respectively).

To demonstrate the efficiency of our proposed method, we show certain examples of the classification results in Figure 9. As shown in this figure, images without a pathological site are accurately classified as negative cases (Figure 9a), while images with pathological sites (small polyp regions appear in Figure 9b, complex vascular structure regions in Figure 9c) are classified as positive cases. However, our proposed method also produces certain incorrect classification results, as shown in Figure 10. In Figure 10a, we show certain sample images that were classified as positive cases (images with pathological sites) even if they did not contain pathological sites. The errors were caused either by the fact that the endoscopic images contain complex vascular structures (the left-most image in Figure 10a) or owing to imperfect input images (middle and right-most images in Figure 10a). In Figure 10b,c, we show certain example images in which our classification method incorrectly classified. As we can observe from these figures, certain text boxes in the input images can cause classification errors (right-most image in Figure 10c). In addition, the input images contain small or blurred polyp regions, resulting in classification errors, which can be solved by super-resolution reconstruction or deblurring in our future works.

**Figure 11:**
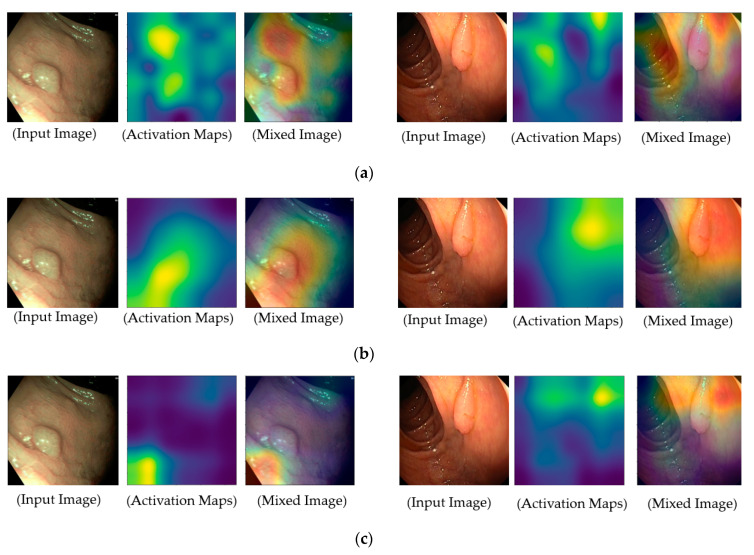
Obtained class activation maps corresponding to the pathological site class using (**a**) VGG-based network, (**b**) inception-based network, and (**c**) DenseNet-based network.

To provide a deep look inside the actual operation of deep learning-based models for classification problems in our study, we measured the regions of focus in the input images on which the classification models are used for accomplishing their functionalities, and the results are illustrated in Figure 11. For this purpose, we obtained the class activation maps using the gradient-weighted class activation mapping (grad-CAM) method [41]. Grad-CAM is a popular method that explains the working of a deep CNN. In the activation maps of these figures, the brighter regions indicate the regions that are focused on in the feature maps, which our system uses for classification purposes. As shown in Figure 11, our deep learning models focus on pathological sites (possible polyps) or complex vascular structures in classifying input images into negative or positive classes. By providing class activation maps to medical doctors, our system can provide a reasonable explanation about why an input image is classified as positive data, and it can demonstrate the functionality of explainable artificial intelligence. In addition, it is a significantly important characteristic of a high-performance classification system in CAD applications.

In our experiments, we use only three sub-models for ensemble algorithm because of two reasons. First, these models (VGG-based, Inception-based, and DenseNet-based) are different in network architecture. Therefore, each sub-model has its own advantages and disadvantages that can be compensated by the other sub-models. Second, although we can use more sub-models to perform ensemble algorithm, the complexity and processing time of the proposed method are increased, which can cause the difficulty in training and deployment of model. Because of these reasons, we only used three sub-models in our experiments. However, it is possible to use more sub-models to possibly enhance the system performance. In that case, our work can be seen as a specific example to demonstrate the enhancement possibility of ensemble algorithm in pathological site classification problem. When the number of sub-models increases, it not only increases the complexity and computation of system, but also represents noises to system that caused by error cases of every individual system.

For a demonstration purpose, we additionally performed experiments with more than three sub-models, i.e., ensemble method with four sub-models. For this purpose, we used an additional CNN classifier based on residual network [23]. The experimental results are given in Table 11. From these experimental results, we see that we obtained a highest classification accuracy of 69.686% using the average rule that is little worse than the accuracy of 70.775% obtained using three sub-models mentioned in Section 3.3. The reason is that when the number of models increases, it also represents noises to the ensemble system caused by error cases of the new model. In addition, the residual network also uses the short-cut connection in the similar way as the DenseNet-based network. Therefore, the residual network shares similar characteristics with DenseNet-based network. Because of these reasons, the performance of ensemble system based on four sub-models is little reduced compared to the case of using three sub-models.

## 4. Conclusions

In this study, we proposed an ensemble learning-based method that combines the classification results of multiple deep learning models to enhance the classification results of the endoscopic pathological site classification system. For this purpose, we first invoked and trained several CNN models, including VGG-, Inception- and DenseNet-based networks for pathological site classification problems. Based on the classification results of these CNN models, we combined them using three combination rules, MAX, AVERAGE and VOTING rules. Using our proposed method, we enhanced the classification performance over that observed in previous studies. In addition, we showed that the AVERAGE rule outperforms the other two combination rules.

## Figures and Tables

**Table 1:** Comparative summaries of proposed and previous studies on image-based polyp or pathological site classification.

Category	Method	Strength	Weakness
Handcrafted feature-based	- Extracts image features using handcrafted image features - Classification based on the extracted image features and classification methods, such as Support Vector Machine (SVM) and k-Nearest Neighbor (k-NN) [11,13]	Easy to implement	- Low accuracy - Only focuses on polyp classification
Deep feature-based	- Trains a single CNN model for classification problem. [9,10,11,13] - Extracts image features using a pretrained convolutional neural network (CNN) and classifies using classification methods such as SVM, k-NN [16]	High accuracy when compared to handcrafted-based method	- More complex than the handcrafted-based method - Requires large amount of training data, strong hardware etc. - Only focuses on polyp classification
	Ensemble of multiple CNNs with different network architectures (Proposed method)	- Focuses on the classification of pathological sites including both polyp and complex vascular information - Extracts rich image features using different architectures of CNN - Combines and takes advantage of single CNN model to enhance classification accuracy	- More complex than previous studies - Requires large amount of training data, strong hardware etc.

**Table 2:** Detail description of the convolutional neural networks (CNNs) used in our study (N/A means “not available”).

Network	Layer	Input Shape	Output Shape	Number of Network Parameter
VGG-based Network	Input Layer	224 × 224 × 3	N/A	0
	Main Convolution Layers in VGG16 Network	224 × 224 × 3	7 × 7 × 512	14,714,688
	Flatten Layer	7 × 7 × 512	25,088	0
	Drop-out Layer	25,088	25,088	0
	Dense Layer	25,088	2	50,178
DenseNet-based Network	Input Layer	224 × 224 × 3	N/A	0
	Main Convolution Layers in DenseNet	224 × 224 × 3	7 × 7 × 1024	7,037,504
	Flatten Layer	7 × 7 × 1024	50,176	0
	Drop-out Layer	50,176	50,176	0
	Dense Layer	50,176	2	100,354
Inception-based Network	Input Layer	224 × 224 × 3	N/A	0
	Main Convolution Layers in Inception network	224 × 224 × 3	5 × 5 × 2048	21,802,784
	Flatten Layer	5 × 5 × 2048	51,200	0
	Drop-out Layer	51,200	51,200	0
	Dense Layer	51,200	2	102,402

**Table 3:** Detailed description of images in the Hamlyn-GI dataset.

Sequence Index	1	2	3	4	5	6	7	8	9	10	Total
Images	705	1003	1700	1349	578	336	493	325	266	1139	7894

**Table 4:** Detailed description of the Hamlyn-GI dataset with the two-fold cross-validation scheme used in our study.

Fold Index		Training Dataset		Testing Dataset		Total
First Fold	Number of Videos	5		5		10
	Number of Images	Without PS	With PS	Without PS	With PS	7894
		2036	3299	1639	920	
Second Fold	Number of Videos	5		5		10
	Number of Images	Without PS	With PS	Without PS	With PS	7894
		1639	920	2036	3299	

**Table 5:** Training parameters used in our study.

Optimizer	Number of Epochs	Initial Learning Rate	Learning Rate Schedule	Batch Size
Adam	30	0.0001	Time decay every epoch	32

**Table 6:** Classification performance using individual CNN-based model (unit: %).

Fold Index	VGG-Based Method			Inception-Based Method			DenseNet-Based Method		
	Sens	Spec	Accuracy	Sens	Spec	Accuracy	Sens	Spec	Accuracy
First Fold	26.304	93.899	69.597	78.370	57.352	64.908	35.978	85.174	67.487
Second Fold	77.417	54.273	68.584	89.967	30.501	67.272	14.974	94.892	45.473
Average	66.271	71.946	68.912	87.438	42.476	66.505	19.554	90.558	52.609

**Table 7:** Classification performance using an individual CNN-based model (unit: %).

Fold Index	VGG-Based Method			Inception-Based Method			DenseNet-Based Method		
	Sens	Spec	Accuracy	Sens	Spec	Accuracy	Sens	Spec	Accuracy
First Fold	14.565	97.010	67.370	22.065	85.723	62.837	27.065	95.729	71.043
Second Fold	62.412	75.491	67.403	6.790	95.138	40.506	13.641	91.601	43.392
Average	51.979	85.088	67.392	10.121	90.938	47.745	16.567	93.442	52.356

**Table 8:** Classification accuracy using our proposed method (unit: %).

Fold Index	MAX Rule			AVERAGE Rule			VOTING Rule		
	Sens	Spec	Accuracy	Sens	Spec	Accuracy	Sens	Spec	Accuracy
First Fold	52.283	82.977	71.942	42.500	88.896	72.215	41.413	88.286	71.434
Second Fold	59.533	63.703	61.124	75.690	61.002	70.084	75.993	59.921	69.859
Average	57.952	72.299	64.630	68.452	73.442	70.775	68.452	72.571	70.369

**Table 9:** Comparison of the classification performances between the proposed method and the previous studies (unit: %).

Method	Accuracy
VGG-based Network [11,25]	68.912
Inception-based Network [26]	66.505
DenseNet-based Network [31]	52.609
Proposed Method with MAX rule	64.630
Proposed Method with AVERAGE rule	70.775
Proposed Method with VOTING rule	70.369

**Table 10:** The comparison of the processing time between the proposed method and the previous studies (unit: ms).

VGG-Based Network [11,25]	Inception-Based Network [26]	DenseNet-Based Network [31]	Proposed Method		
			Preprocessing Step	Ensemble of Three CNNs	Total
37.646	67.472	65.901	8.421	171.019	179.440

**Table 11:** Classification accuracy using our proposed method with four sub-models (unit: %).

Fold Index	MAX Rule			AVERAGE Rule			VOTING Rule		
	Sens	Spec	Accuracy	Sens	Spec	Accuracy	Sens	Spec	Accuracy
First Fold	57.500	70.165	65.612	54.783	77.181	69.129	55.978	75.290	68.347
Second Fold	59.867	63.114	61.106	82.904	48.969	69.953	79.691	53.978	69.878
Average	59.351	66.286	62.567	76.772	61.551	69.686	74.520	63.483	69.382
